# Clinical profile of *Plasmodium falciparum* and *Plasmodium vivax* infections in low and unstable malaria transmission settings of Colombia

**DOI:** 10.1186/s12936-015-0678-3

**Published:** 2015-04-11

**Authors:** Myriam Arévalo-Herrera, Mary Lopez-Perez, Luz Medina, Alberto Moreno, Juan B Gutierrez, Sócrates Herrera

**Affiliations:** Caucaseco Scientific Research Center, Cali, Colombia; Faculty of Health, Universidad del Valle, Cali, Colombia; Asoclinic Inmunología Ltda, Cali, Colombia; Emory University, Atlanta, GA USA; Department of Medicine, Division of Infectious Diseases, Emory University, Atlanta, GA USA; University of Georgia, Athens, GA USA

**Keywords:** Clinical profile, *Plasmodium falciparum*, *Plasmodium vivax*, Malaria, Colombia

## Abstract

**Background:**

Malaria transmission in Latin America is generally hypoendemic and unstable, with *Plasmodium vivax* as the most prevalent species. However, only a few studies have been carried out in areas with low and unstable transmission, whereas the clinical profile of malaria has been broadly described in hyperendemic areas. The pattern of clinical manifestations and laboratory findings in low to moderate endemic areas of Colombia is reported here.

**Methods:**

A passive surveillance study was conducted between 2011 and 2013 involving 1,328 patients with *Plasmodium falciparum*, *P. vivax* or mixed malaria infections attending malaria points-of-care of four malaria endemic-areas with distinct transmission intensities and parasite distribution. Trained physicians recorded clinical symptoms and signs as well as socio-demographic characteristics of study participants. Haematological, biochemical and urine tests were performed at the time of diagnosis.

**Results:**

Out of 1,328 cases, 673 (50.7%) were caused by *P. vivax;* 650 (48.9%) were due to *P. falciparum*; and five (0.4%) patients had mixed infections (*P. falciparum/P. vivax*). Most patients (92.5%) presented with uncomplicated malaria characterized by fever, chills, headache, sweating, myalgia/arthralgia and parasitaemia ≤ 20,000 parasites/μL. Fever, tachycardia, pallor and abdominal pain on palpation were more frequent in *P. falciparum* patients, whereas mild hepatomegaly and splenomegaly were mostly observed with *P. vivax*. Non-severe anaemia (Hb 7.0-10.9 g/dL) was observed in 20% of the subjects, whereas severe anaemia (Hb < 7.0 g/dL) was present in four patients. Half of the patients presented thrombocytopaenia regardless of parasite species. Leukopaenia, neutrophilia and monocytosis were frequently observed in patients infected with *P. falciparum*. Mild-to-moderate biochemical alterations were present in ~25% of the patients, particularly abnormal bilirubin in those with *P. falciparum* and abnormal transaminases in *P. vivax* malaria patients. Proteinuria was present in ~50% of the patients regardless of parasite species, whereas haemoglobinuria was more common in *P. vivax* infections. Only 7.5% of the cases were classified as clinically severe malaria, caused by both *P. vivax* and *P. falciparum*.

**Conclusions:**

The high prevalence of uncomplicated malaria associated with moderate parasitaemia suggests the importance of timely diagnosis and effective treatment and encourages new activities to further decrease complicated malaria cases and mortality.

## Background

Malaria remains a major public health problem in the developing world. In 2013, it caused an estimated 198 million clinical cases and 584,000 deaths [1]. The vast majority of these cases (80%) and deaths (90%) were caused by *Plasmodium falciparum* infections in sub-Saharan Africa, the region with the highest rates of transmission worldwide. High frequency of severe disease and fatal complications occur primarily during infancy and early childhood in this region [1,2]. *Plasmodium vivax* is the second most prevalent species that accounts for 75 to 85 million cases/year, corresponding to >50% of malaria infections outside of Africa [3]. In Latin America, malaria transmission is typically classified as hypoendemic and unstable (annual parasite incidence, API < 0.1 per 1,000 per year) for both *Plasmodium* species, with ~145 million inhabitants at risk of infection [1,4,5] and ~469,000 cases reported in 2012, equivalent to ~0.3% of the global burden. A total of 108 deaths reported in the region in 2012 correspond to ~0.02% of the reported fatal malaria cases worldwide; the Amazon basin generating ~90% of the malaria cases [1]. Colombia accounts for ~13% of the malaria cases [1,6], predominantly by *P. vivax* (>70%), which co-exists with *P. falciparum*, with a remarkably different regional prevalence [6,7]. Such differences in prevalence are mainly due to the varied Duffy antigen expression in these populations [8,9], and the clinical relapses due to *P. vivax* hypnozoite activation [10].

Human malaria has a broad clinical spectrum that includes asymptomatic infection, uncomplicated malaria, and complicated and lethal malaria cases [11]. This clinical spectrum depends on the complex interaction between the parasite, human host and environmental factors [12]. In temperate and sub-tropical regions of Asia and Latin America, residents of all ages have low levels of naturally acquired immunity, and thus typically present with acute or severe disease to mild and more chronic infections, particularly in adult men [2,13]. Most of the studies designed to better understand the association of the clinical profile of malaria with the parasite species have been carried out in areas of high malaria endemicity in Africa and Asia (e.g. Papua New Guinea and Indonesia), whereas studies carried out in low-to-moderate endemic areas around the world, particularly in Latin America, are scarce. Although Colombia is one of the major contributors to malarial morbidity and mortality in the region, limited information about the clinical profile and malaria severity is available [14-18], and much is based on retrospective studies of hospitalized subjects conducted before 2008 [19-21]. In those reports, severe anaemia, severe thrombocytopenia and hepatic failure were the most frequently reported complications [14-18,20,21].

During the last decade, Colombia has experienced a malaria-decreasing trend [1,22], which could be explained by greater efforts to reduce disease burden, severity and mortality due to extensive use of insecticide bed-nets, and early diagnosis and prompt treatment. In addition, the low number of severe cases might also be due to the introduction of explicit indication of clinical signs of severe malaria in the Colombian Ministry of Health and Social Protection (MoH) guidelines [19,23,24]. A recent study reported that at least for the regions studied, the malaria point-of-care (POC) were all located within an hour away from the patients households [25], which greatly contributed to early diagnosis and treatment with a consequent reduction of complicated cases and mortality [7,19]. It appears that early consultation reduces the contact of health care personnel in endemic areas with severe malaria cases, as most of these are likely to be treated in larger cities with greater health care infrastructure. Although this may benefit patients, it also leads to a dispersion of complicated cases with potential loss of valuable epidemiological information to accurately assess risk factors for morbidity and mortality. This study aimed at prospectively collecting information on the clinical profile of malaria from a large number of subjects acutely infected with *P. falciparum* and *P. vivax* residing in some of the highest malaria transmission regions in Colombia.

## Methods

### Study design and ethical issues

A passive surveillance study was conducted between 2011 and 2013 in four malaria outpatient clinics located in areas with distinct transmission intensity and parasite distribution. A total of 1,328 patients presenting malaria related symptoms were passively recruited at malaria POC. One site (Buenaventura) was excluded from the study since September 2012 because of the relatively low number of malaria cases. Patients with malaria infections confirmed by microscopic examination of Giemsa-stained thick blood smears (TBS) [26], and who received oral and written information about the study before enrollment, were asked to provide a written informed consent (IC) or an informed assent (IA) in the case of children <18 years/old. Both documents were previously approved by the Institutional Review Board (IRB) affiliated with the MVDC (Malaria Vaccine and Drug Development Center, CECIV). A trained physician on the study research staff completed a standard clinical evaluation of all malaria symptomatic subjects. The local health provider treated all individuals as soon as the blood sample was drawn, using the national protocol for malaria treatment [23]. Patients infected with *P. vivax* were treated orally with curative doses of chloroquine (25 mg/kg provided in three doses) and primaquine (0.25 mg/kg daily for 14 days), whereas *P. falciparum* patients received artemether plus lumefantrine (six doses over three days). Each individual received a unique numerical code to simplify data collection and identification.

### Study sites

**Figure 1:**
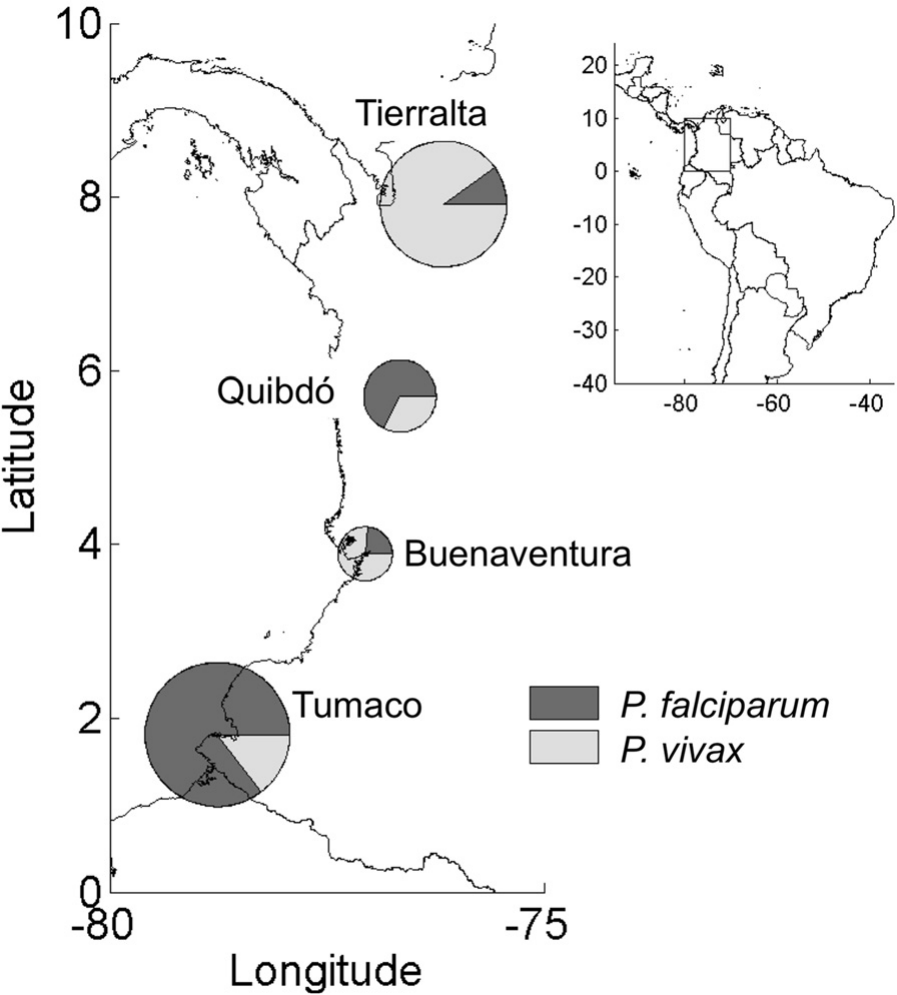
Distribution of malaria cases in the study areas. The charts display the proportion of *Plasmodium* species per site. *P. vivax* cases were more frequent in Tierralta (90%) and Buenaventura (77%), whereas *P. falciparum* infections were more prevalent in Quibdó (66%) and Tumaco (85%).

Four defined endemic settings in Colombia were selected because of the relatively high level of malaria prevalence: Tierralta (Department of Córdoba); Quibdó (Department of Chocó); Tumaco (Department of Nariño); and Buenaventura (Department of Valle del Cauca) (Figure 1). *Plasmodium vivax* and *P. falciparum* are both transmitted in different proportions in these respective regions, which display an unstable endemic pattern [6,7]. Tierralta has a population of ~90,000 inhabitants with 44.4% living in rural areas. Most of inhabitants are described as mestizo ethnicity with a small Amerindian Emberá Katío indigenous community. The predominant malaria parasite species in this region is *P. vivax* (~85%). Tumaco is a port city situated in the Pacific Coast close to the Ecuadorian border with a population of ~160,000 inhabitants, predominantly Afro-descendants with an Amerindian Awá indigenous community. The predominant malaria parasite species in the region is *P. falciparum* (~79%). Quibdó is situated on the Pacific Coast of Colombia close to the border with Panamá, with a population of ~100,000, mainly Afro-descendants. Most malaria cases in Quibdó are caused by *P. falciparum* (~70%). Buenaventura has a population of 350,000 inhabitants with >90% living in the urban area. Most of the inhabitants are Afro-descendants and mestizos; in this site *P. vivax* is the most prevalent malaria species (~75%).

### Clinical assessment

Patients were classified as uncomplicated or severe malaria cases according to the WHO clinical and laboratory criteria [27] and the Colombian MoH guidelines [23]. The latter are more conservative in some definitions based on previous evidence: anaemia (Hb < 7 g/dL in adults and children), renal dysfunction (serum creatinine > 1.5 mg/dL), severe thrombocytopenia (≤20,000 platelets/μL) and hyperparasitaemia (>50,000 parasites/μL). Uncomplicated malaria was defined as a clinical malaria case: symptoms such fever >38°C, headache, chills and/or malaise and a positive TBS without severity criteria, regardless of parasite species. Severe malaria was defined as one or more of the clinical or laboratory parameters [27,23], regardless of the malaria parasite species. Clinical and parasitological signs of prognostic danger were recorded [24].

### Laboratory tests

Whole blood (7–15 mL) was drawn by venipuncture at the time of enrollment before anti-malarial treatment. Samples were collected into Vacutainer tubes containing either EDTA for plasma collection, or clot activator and gel for serum separation. Giemsa stained TBS [26] were examined by a trained malaria microscopists who determined parasite species and density, which were expressed in parasites/μL after counting the number of parasites per 200 white blood cells (WBC); counts were normalized using the actual WBC counts for each patient. For quality control, 10% of TBS were re-examined by a second microscopist and all samples were confirmed by real-time quantitative PCR (RT-qPCR) as described elsewhere [28].

Complete blood cell counts were performed using an automated haematology analyzer (KX-21 N, Sysmex, Japan) and both urine dipstick and urine microscopic analyses were carried out at the local health facility. Sera for blood chemistry profiles were stored frozen and analysed using commercial kits at the central Asoclinic laboratory in Cali (non-endemic area). The following parameters were measured: haemoglobin (normal values 11.0-16.5 g/dL), haematocrit (normal value 35-50%), creatinine (normal values 0.6-1.1 mg/dL in females, and 0.7-1.4 mg/dL in males), blood urea nitrogen (BUN; normal values 7–21 mg/dL), glycaemia (normal values 60–110 mg/dL), total bilirubin (TB; normal values ≤ l.0 mg/dL), direct bilirubin (DB; normal values ≤ 0.25 mg/dL), alanine aminotransferase (ALT; normal values ≤ 31 U/L in females, and ≤ 40 U/L in males), aspartate aminotransferase (AST; normal values ≤ 32U/L in females and ≤ 38U/L in males).

### Statistical analysis

Study data were collected and managed using REDCap (Nashville, Tennessee, USA) with electronic data capture tools [29]. Data were analysed with the statistical software MATLAB® 2013a (The MathWorks, Inc., Natick, Massachusetts, USA). Nominal variables were analysed using descriptive statistics. The Mann–Whitney *U* test was used to compare two groups. Spearman’s rank correlation (r_s_) was used to assess the correlation between numeric variables. Chi-square or Fisher’s exact test were used to compare proportion differences. A p-value < 0.05 was considered statistically significant.

## Results

### Demographic data and malaria history

A total of 1,328 subjects (55.4% male) with acute malaria were enrolled in four endemic sites between 2011 and 2013. A total of 563 patients were recruited in Tierralta, 549 in Tumaco, 177 in Quibdó, and 39 in Buenaventura (Figure 1). *Plasmodium* species distribution was statistically different in the study sites; however the overall distribution indicated a total of 673 (50.7%) participants presenting with *P. vivax* malaria, and 650 (48.9%) with *P. falciparum* infections. Mixed malaria infections (*P. vivax/P. falciparum*) were found in five cases (0.4%); four in Quibdó and one in Buenaventura.

**Figure 2:**
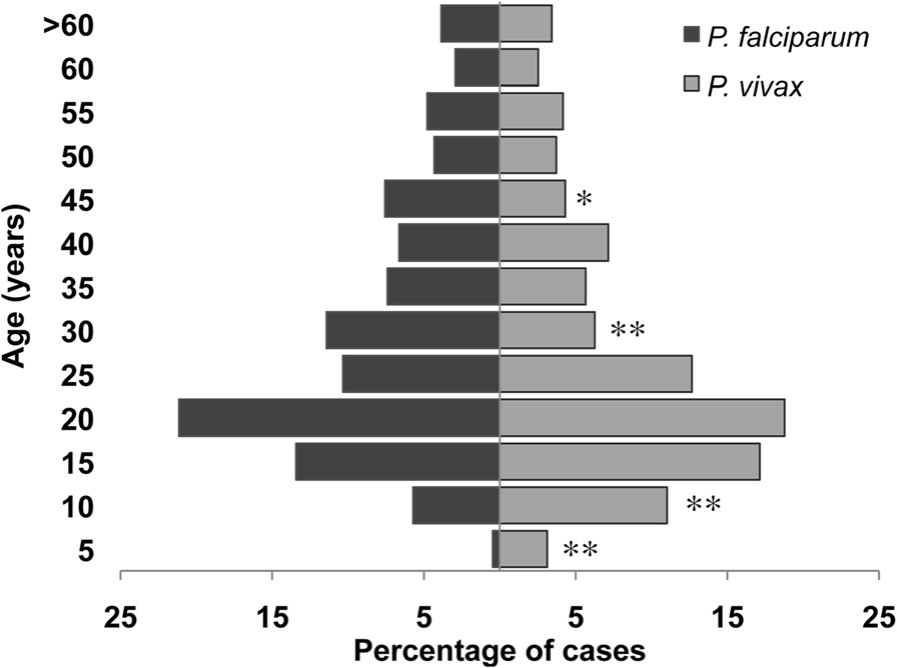
Prevalence and parasite species distribution according to age. Percentage of subjects infected with either *P. falciparum* or *P. vivax* parasites at each stratified age group are shown. Statistical differences between *P. falciparum* and *P. vivax* infections were calculated using the Chi-square test. *p value < 0.05, **p value < 0.01.

Patients had a mean age of 26 years (median 21 years; IQR 14–36 years); children ≤ 15 years of age (25.5%) were mainly infected with *P. vivax*, whereas young adults between 16 and 30 years of age (40.3%) were infected with either *P. vivax* or *P. falciparum* (Figure 2). Some differences in race and occupation were observed between *P. falciparum* and *P. vivax* infected patients. Tumaco malaria cases were mostly due to *P. falciparum* where population is mainly Afro-descendent dedicated to fishing, whereas in Tierralta where population is mainly mestizo farmers, most cases were induced by *P. vivax* (Table 1, Figure 1). Differences in some demographic and epidemiological variables were observed between *P. falciparum* and *P. vivax* infected patients (Table 1). Patients presenting mixed infections (n = 5, 3 males) were 8 to 37 years old and reported a median of 8 days of illness (range 3 to 17 days).

**Table 1:** **Demographic data and malaria history per parasite species**

		***P. falciparum*** **(n = 650)**		***P. vivax*** **(n = 673)**		**p value** ^***b***^
		**Median**	**IQR** ^***a***^	**Median**	**IQR**	
Age (years)		**24**	16-38	19	12-33	**<0.001**
Time of residence (years)		4	1-4	4	3-4	0.365
Previous malaria episodes		2	1-3	**3**	1-5	**<0.001**
Days of illness		4	2-6	4	3-5	0.689
**Frequencies**		**n**	**%**	**n**	**%**	**p value** ^***c***^
Male		334	51.4	**399**	**59.3**	**0.004**
Race						
	Afro-descendant	**526**	**80.9**	230	34.2	**<0.001**
	Mestizo	91	14.0	**415**	**61.7**	**<0.001**
	Indigenous	28	4.3	22	3.3	0.322
Occupation						
	Student	237	36.5	226	33.6	0.272
	Housewife	**177**	**27.2**	150	22.3	**0.037**
	Farmer	47	7.2	**141**	**21.0**	**<0.001**
	Other^*d*^	189	29.1	155	23	0.251
Previous malaria episodes (YES)		222	34.2	**452**	**67.2**	**<0.001**
Malaria episodes in the last year		65	29.2	**232**	**51.3**	**<0.001**
Anti-malarial treatment^*e*^		43	19.4	314	**69.5**	**<0.001**

^*a*^IQR: interquartile range. ^*b*^p value using Mann–Whitney test between *P. falciparum* and *P. vivax*. ^*c*^p value using the Chi-square test between *P. falciparum* and *P. vivax*. ^*d*^fisherman, miner, timber exploitation, other. ^*e*^treatment for the last malaria episode. Most frequent and significant data are highlighted in bold.

Of the entire study population, 677 subjects (51.0%) self-reported to have experienced previous lifetime malaria episodes with a median of two previous episodes. A majority of patients (n = 425; 62.8%) reported having suffered malaria within the last six months prior to current episode, and 6.2% of these during the last month, while 2.3% reported ≥ 10 lifetime episodes.

A total of 359/677 (53%) patients who reported previous malaria episodes, declared to have received anti-malarial chemotherapy for the last malaria episode, and most (~80%) reported taking the complete treatment regimen. Overall, patients presented at health facilities promptly for malaria diagnosis regardless of parasite species, although a few patients (n = 42) reported >15 days of illness. A low proportion of patients (n = 77) self-reported concomitant clinical entities such as chronic cardiovascular diseases (20/77), gastrointestinal disorders (13/77), chronic respiratory diseases (11/77), diabetes (2/77) and others.

Most patients (97%) presented with low-to-moderate parasitaemia (≤20,000 parasites/μL), with the median parasitaemia significantly higher for *P. vivax* (3,314 parasites/μL; IQR 1,486-6,521) than for *P. falciparum* (1,482 parasites/μL; IQR 602–3,782).

### Uncomplicated malaria

#### Clinical findings

**Figure 3:**
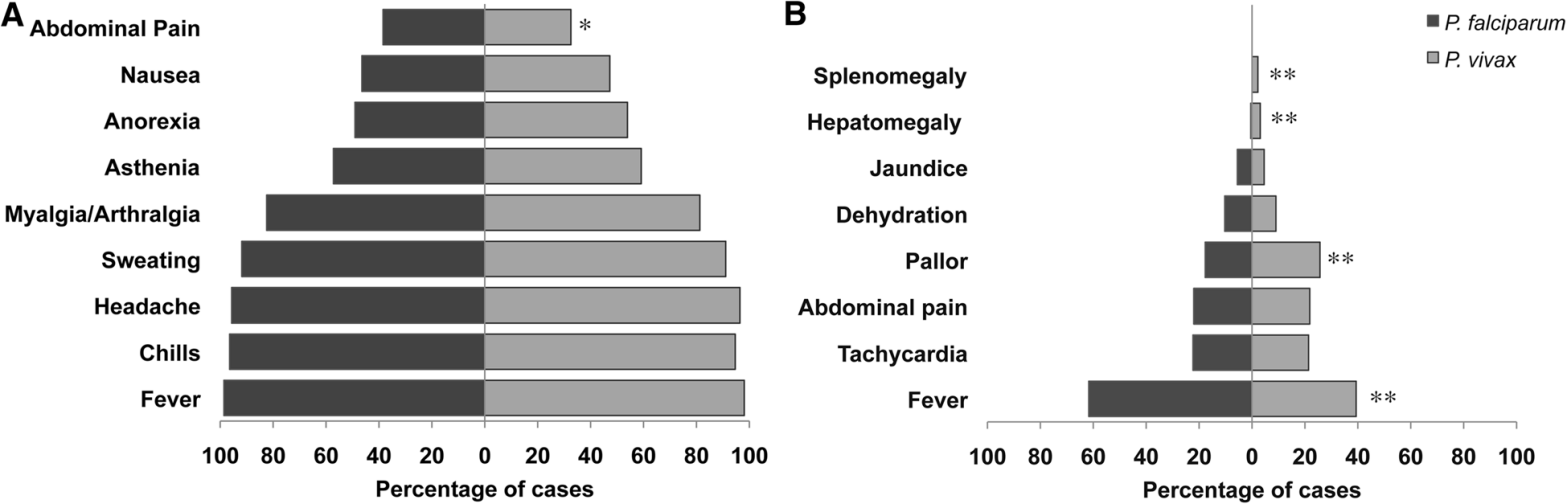
Frequency of symptoms and signs associated with *P. falciparum* and *P. vivax* infections in the study population. Data shown are the percentages of malaria patients that reported every symptom (**A**) or presented with the listed signs (**B**). All patients reported more than one symptom or had more than one sign. Statistical differences between *P. falciparum* and *P. vivax* infections were calculated using the Chi-square test. *p value < 0.05, **p value < 0.01.

A total of 1,229 individuals (92.5%) were classified as uncomplicated malaria, and >90% reported with the classical malaria triad of fever, chills and sweating, together with headache (Table 2, Figure 3). Overall, symptoms presented with a similar distribution for both parasite species (Figure 3A). Most frequent clinical signs on physical examination are shown in Figure 3B. Fever (axillary temperature >38°C) was significantly more frequent in *P. falciparum* cases, whereas pallor was more frequent in *P. vivax* infected patients (Figure 3B). Uncommon symptoms, such as vomiting (23%), cough (15%) and diarrhea (8%), were mostly reported in *P. vivax* patients. Hyperpyrexia (8.1%) and hypothermia (1.3%) were also observed. Ten patients presented with hepatosplenomegaly (ranged 1–3 cm below the costal margin) all with *P. vivax* infections; one had a mixed infection. None presented with hepatic dysfunction, although mild alterations in total bilirubin and hepatic enzymes were observed.

**Table 2:** **Clinical profile of all patients studied**

**Classification**		***P. falciparum***	**n = 650**	***P. vivax***	**n = 673**	**p value** ^***a***^
		**n**	**%**	**n**	**%**	
Uncomplicated malaria		529	81.4	566	84.1	0.191
	Warning signs	68	10.5	61	9.1	0.392
Complicated malaria						
	One complication	43	6.6	42	6.2	0.781
	Two complications	10	1.5	4	0.6	0.093

^*a*^p value using the Chi-square test between *P. falciparum* and *P. vivax*.

#### Haematological findings

Thrombocytopenia was rather common (48.6%) although in most cases it was mild-to-moderate (50,000 to 150,000 platelets/μL); only 20 patients (1.5%) had low platelets levels (13,000 to 50,000 platelets/μL), with a negative correlation between platelets count and parasitaemia (r_s_ = −0.167; p < 0.0001). Anaemia presented in 19.7% of patients, mostly ≤ 15 years of age (60.1%; p < 0.0001) with only three patients (0.2%) displaying severe anaemia. Neither thrombocytopenia nor anaemia was associated with parasite species, and more patients infected with *P. vivax* had lower erythrocyte mean corpuscular volumes (16.3% *vs* 9.9%) and mean corpuscular haemoglobin concentration (54.0% vs 6.9%) than those infected with *P. falciparum*. Leukocyte alterations, including leukopaenia (27.3%), neutrophilia (60.8%) and monocytosis (21.3%) were more common in *P. falciparum* infections (p < 0.01) and displayed a positive correlation between leukocyte count and parasitaemia (r_s_ = 0.348; p < 0.0001), but none presented with severe deviations.

### Clinical biochemical findings

Blood chemistry parameters of hepatic and renal function were normal in >60% of the patients. Mild-to-moderate alterations in bilirubin levels [TB (11%), DB (16%)] were found mainly in patients infected with *P. vivax* (p < 0.01), with a positive correlation between TB levels and parasitaemia (r_s_ = 0.155; p < 0.0001). In contrast, abnormal transaminases (ALT and AST) from mild-to-moderate levels were more frequent in *P. falciparum* patients than *P. vivax* (14% *vs* 8%; p < 0.05) (Table 3). Renal function parameters showed mild alterations in creatinine (26%) and BUN (5%), with similar distribution between parasites species. Proteinuria was observed in 54% *P. falciparum* patients and 47% with *P. vivax* (p = 0.014), and it was associated with creatinine levels >1.5 mg/dL (p = 0.013). Other significant alterations more frequently found in *P. falciparum* than in *P. vivax*-infected patients (p < 0.001), were haematuria (26% *vs* 4%), ketonuria (22% *vs* 12%) and leukocyturia (17% *vs* 4%), respectively. In contrast, haemoglobinuria was more frequently observed in *P. vivax* infections (14% *vs* 4%; p < 0.001). Hypoglycaemia, ranging between 46 and 59 g/dL was found only in four patients.

**Table 3:** **Clinical laboratory parameters**

**Laboratory parameters**	**Uncomplicated malaria (n = 1225)**		**p value** ^***b***^	**Severe malaria (n = 99)**		**p value** ^***b***^
	**Median (IQR** ^***a***^ **)**			**Median (IQR** ^***a***^ **)**		
	***P. falciparum***	***P. vivax***		***P. falciparum***	***P. vivax***	
Haemoglobin (g/dL)	12.5 (11.2-13.6)	12.7 (11.3-14)	**0.024**	12.8 (11.5-13.8)	12.8 (10.9-14.2)	ns
Platelets count (x10^3^/μL)	159 (123.5-209)	**150 (112–196.5)**	**0.008**	120 (83–159)	138 (82–660)	ns
Total bilirubin (mg/dL)	0.6 (0.3-0.9)	**0.7 (0.4-1.1)**	**<0.001**	1.0 (0.3-2.5)	1.0 (0.5-3.4)	ns
Direct bilirubin (mg/dL)	0.17 (0.08-0.27)	**0.18 (0.10-0.32)**	**0.002**	0.25 (0.10-0.98)	0.32 (0.18-0.52)	ns
ALT (U/L)	**33 (24–48)**	29 (23–38)	**<0.001**	**50 (19–141)**	19 (15–38)	**0.001**
AST (U/L)	**21 (14–32)**	17 (12–24)	**<0.001**	**75 (32–154)**	33 (24–50)	**0.001**
Creatinine (mg/dL)	**1.0 (0.9-1.2)**	0.9 (0.8-1.1)	**<0.001**	1.0 (0.9-1.3)	1.0 (0.8-1.3)	ns

^*a*^IQR, interquartile range; ALT, Alanine aminotransferase; AST, aspartate aminotransferase; ns, not significant. ^*b*^p value using the Mann–Whitney test between *P. falciparum* and *P. vivax*. Most frequent and significant data are highlighted in bold.

### Malaria with warning signs

A total of 129/1,323 patients (9.8%), excluding five with mixed infections, presented with one or more signs suggesting severe malaria development [24] (Table 4). Tachypnea in absence of fever was the most frequent. Twenty-four patients (1.8%) presented with persistent vomiting (range 5 to 15 episodes), and six presented with signs of dehydration. Few patients (14/1,323) had consciousness alteration including somnolence (11/14), lethargy or irritability and aggressiveness (3/14). A child (12 years old) presented with only one episode of generalized seizures (<1 min) without any other complication related to cerebral malaria.

**Table 4:** **Warning signs of malaria in the study population**

**Sign**	***P. falciparum*** **(n = 68/650)**		***P. vivax*** **(n = 61/673)**		**p value** ^***a***^
	**n**	**%**	**n**	**%**	
Tachypnea without fever	17	2.6	30	4.5	0.076
Choluria	**30**	**4.6**	13	1.9	**0.007**
Persistent vomiting	11	1.7	13	1.9	0.744
Consciousness alteration	9	1.4	5	0.8	0.254
Convulsions	1	0.2	0	0	NA

^*a*^p value using the Chi-square test. NA, not applicable. Most frequent and significant data are highlighted in bold.

Choluria (bilirubin in urine) was associated with *P. falciparum* infection, microscopic haemoglobinuria (Hb-positive dipstick) and proteinuria (p < 0.010), suggesting an important renal involvement. Microscopic haematuria (>3 RBC) and hyperbilirubinaemia (TB >3 mg/dL) were also observed in 28% and 12% of patients, respectively, with choluria.

### Severe malaria

A total of 99 patients (57% male) with a mean age of 27 years (median 23 years; IQR 15–38 years) were classified with complicated malaria (Table 5). Fifty-four (4.1%) of which required hospitalization because of their clinical condition. Thirteen of these patients presented with two complications simultaneously, one of which was hyperparasitaemic; a total of 41 patients had only one complication (Table 2). The most frequent complications in patients infected with either *P. vivax* or *P. falciparum* were: severe prostration (n = 11), respiratory distress (n = 4), hyperparasitaemia (n = 4), abnormal spontaneous bleeding (n = 4), severe anaemia (n = 4), cardiogenic shock (n = 1), and severe thrombocytopenia (n = 1) as well as malaria warning signs including tachypnea in absence of fever and persistent vomiting or diarrhoea. Cerebral malaria was not observed. The remaining 45 patients were diagnosed and treated at the POC as uncomplicated malaria cases; however, when laboratory test were analysed, these revealed abnormalities indicating hepatic and renal dysfunctions according to the WHO and Colombian MoH guidelines [23,27]. However, whether some of these cases were diagnosed as complicated malaria in other health care institutions after the study enrollment, could not be confirmed.

**Table 5:** **Frequency of complications in malaria patients**

**Criteria**	***P. falciparum***	***P. vivax***	**p value** ^***a***^
	**n (%)**	**n (%)**	
**One complication**	**43**	**42**	
Hepatic dysfunction (TB >3 mg/dL)	8 (15.1)	12 (26.1)	ns
Hepatic dysfunction (ALT > 120 U/L)	**12 (22.6)**	2 (4.3)	**0.001**
Renal dysfunction (serum creatinine > 1.5 mg/dL or BUN > 40 mg/dL)	7 (13.2)	7 (15.2)	ns
Prostration	6 (11.3)	5 (10.9)	ns
Haemoglobinuria (urine dipstick)	1 (1.9)	**9 (19.6)**	**0.005**
Respiratory distress	1 (1.9)	3 (6.5)	ns
Hyperparasitaemia (>50,000 asexual parasites/μL)	3 (5.7)	1 (2.2)	ns
Abnormal spontaneous bleeding	3 (5.7)	0 (0.0)	NA
Severe anaemia (Hb < 7 g/dL)	1 (1.9)	2 (4.3)	ns
Cardiogenic shock/systolic dysfunction (SBP < 70 mm Hg)	1 (1.9)	0 (0.0)	NA
Severe thrombocytopaenia (≤20,000 platelets/μL)	0 (0.0)	1 (2.2)	NA
**More than one complication**	**10**	**4**	
Hyperparasitaemia plus			
Severe thrombocytopaenia	2 (3.8)	0 (0.0)	NA
Hepatic dysfunction	1 (1.9)	1 (2.2)	ns
Renal dysfunction	1 (1.9)	0 (0.0)	NA
Respiratory distress	1 (1.9)	0 (0.0)	NA
Severe anaemia	1 (1.9)	0 (0.0)	NA
Haemoglobinuria	0 (0.0)	1 (2.2)	NA
Hepatic dysfunction plus			
Prostration	2 (3.8)	0 (0.0)	NA
Severe thrombocytopaenia	1 (2.2)	0 (0.0)	NA
Renal dysfunction	1 (1.9)	0 (0.0)	NA
Renal dysfunction plus			
Prostration	0 (0.0)	1 (2.2)	NA
Abnormal spontaneous bleeding	1 (1.9)	0 (0.0)	NA

^*a*^p value using the Fisher’s exact test. ALT, alanine aminotransferase; BUN, blood urea nitrogen; Hb, haemoglobin; NA, not applicable; ns, not significant; SBP, systolic blood pressure; TB, total bilirubin. Most frequent and significant data are highlighted in bold.

Hepatic dysfunction with alteration in hepatic enzymes was more frequent in *P. falciparum* infections and observed only in adults. In contrast, haemoglobinuria detected by urine dipstick in absence of microscopic haematuria was most frequent in adults infected by *P. vivax*. Renal dysfunction following the WHO criteria was not reported, but 14 patients met the Colombian MOH criteria (serum creatinine > 1.5 mg/dL). This alteration was more frequent in adults than children < 15 years of age (18% *vs* 4%). In patients with complicated malaria anaemia (23.2%), leukopenia (32.3%), and thrombocytopenia (64.6%) were common haematological alterations. Abnormal transaminases (ALT and AST) and hyperbilirubinaemia (TB > 1 mg/dL) were also observed in 45.5% and 46.5% of patients, respectively (Table 2).

## Discussion

As expected most malaria cases reported at the study sites had no clinical complications (92.5%), which appear to correlate with the relatively low malaria transmission in the study areas [7], together with the early diagnosis and prompt treatment recently reported [30]. During the last decade the MoH has been expending considerable effort in an attempt to reduce the burden of infection and to eliminate malaria mortality. Additionally since 2005, the Global Fund for AIDS, tuberculosis and malaria (GFATM) has sponsored reinforcement of control activities in most endemic areas of Colombia, including the three study sites described here. This GFATM project appears to have significantly contributed to a reduction in malaria transmission and, therefore, to the low mortality reported, particularly in areas of easy access to health care [19,22]. The fact that it was found that 4.1% (54/1,328) of the malaria cases attending the POC required hospitalization even though most (41/54) displayed only a single severity criterion indicates a great underestimation in the number of complicated malaria cases in official reporting. Between 2011 and 2013, a total of 1,322 of the ~172,000 malaria cases (0.8%) reported in Colombia during that period were classified as complicated cases; 48 deaths (~0.3 per thousand) were considered malaria-related (National Surveillance Service of Colombia, SIVIGILA). This underestimation is also supported by the 45 individuals managed as uncomplicated malaria at the POC, who were later found to have clinical laboratory parameters compatible with the severe malaria case definition [23,27]. Patients attending the malaria POC are usually evaluated by community health workers, with low level of education, supervision and training, especially for clinical evaluation; whereas in this study a trained physician evaluated all patients.

Likewise, signs associated with severe clinical malaria emphasized in the MoH guidelines (warning signs) have significantly contributed to the early identification of potential malaria complications and to a decrease in the risk of severity and mortality. Warning signs reported here in 129 patients, including choluria, persistent vomiting and diarrhoea, tachypnea in absence of fever, hyperpyrexia and others, have been reported in previous clinical studies conducted in Colombia [16,24,31].

In contrast, in countries with higher malaria transmission such as Papua New Guinea [32] and Indonesia [33], the severe malaria incidence of individuals attending health facilities was 6.2% and 9%, respectively. Studies about prevalence of severe malaria in Latin America are scarce and there have been no previous studies for the identification of severe cases at primary health facilities. Available information consists only of a series of case reports of hospitalized patients [20,21,34-37]. Interestingly, there has been a surge in studies reporting the contribution of *P. vivax* to severe malaria burden, challenging the idea that vivax malaria is ‘benign’ and not lethal [38,39]. Moreover, there have been recent reports on the role of *P. vivax*-infected erythrocytes adherence and pathophysiology of *P. vivax* [40-42]. In fact, several *P. vivax* severe cases there have been reported in Colombia [21], Brazil [34,35], Perú [36] and Venezuela [37], in agreement with this study.

Severe anaemia significantly contributes to malaria mortality [43]. As in previous reports [16,14,44], anaemia was only present as mild-to-moderate in ~20% of the cases, and severe in only four patients. Because there is an inverse correlation between Hb levels and days of illness [45], results would support a short duration of infection and possibly the presence of iron deficiency as previously reported [46]. While most patients had a low-to-moderate parasitaemia, higher median parasitaemia was found in *P. vivax*-infected patients (3,314 parasites/μL) than in those infected with *P. falciparum* (1,482 parasites/μL). This finding is in agreement with previous reports in Colombia [16] and may be explained by the lower age, and consequently less cumulative malaria exposure in the group most infected by *P. vivax* (children ≤ 15 years/old), which had a significantly higher parasitaemia than the older age group.

Although thrombocytopaenia has been reported in up to 94% of acute malaria cases, frequently with severe intensity [47], here it was observed in less than half of the cases, with levels that did not represent a risk of spontaneous bleeding. Both thrombocytopaenia and anaemia are multifactorial but in both cases immunological factors have been considered important. The presence of anti-platelet antibodies as well as phagocyte-mediated platelet clearance have been previously described in Colombia and Brazil [48,49], however this hypothesis was not tested in this study.

Malaria-induced changes in the leukocyte populations are very diverse [50-54]; most commonly described alterations like mild-to-moderate leukopaenia, neutrophilia and monocytosis were transiently observed in this study. Leukocytosis, previously associated with severe disease and considered of poor prognosis [53], was not reported here.

Because a significant proportion of the malaria cases (40.3%) occurred in the economically active population (16–30 years of age), and lower prevalence (25.5%) was found in children ≤ 15 years of age, the selection of the study populations was not biased and it is speculated that malaria transmission may occur near to or inside households and schools where children ≤ 15 years old spend most of their time. Although adult members of the family also spend a significant amount of time in the house and surrounding neighborhood, they did not appear to greatly contribute to malaria cases in this study, possibly harboring asymptomatic infections. A recent study in the same sites has indicated a prevalence of ~6% of asymptomatic carriers. In addition, semi-immune young adult volunteers (≥18 years old) from these sites, when exposed to experimental challenge with infectious *P. vivax* sporozoites developed significantly fewer symptoms than naïve volunteers [55]. In this context it could also be hypothesized that the relatively high prevalence of uncomplicated malaria in this study could be related to a significant level of clinical immunity, given the long-term residence of subjects in the endemic area, as was observed previously in Colombia [55]. In low transmission settings malaria clinical immunity develops significantly later; however, the previous study showed that five to 10 years of exposure at low transmission intensity could induce significant clinical immunity. In fact, 50% of patients reported living in the study areas >5 years with 51% reporting multiple previous malaria episodes. Furthermore, the almost equal proportion of *P. vivax* and *P. falciparum* cases appears to be due to the fact that most of the population where *P. vivax* is prevalent are mestizos, whereas *P. falciparum* was more prevalent in Afro-descendants with high prevalence of Duffy negative blood group [8,9].

## Conclusions

The present study showed a high prevalence of uncomplicated malaria together with moderate parasitaemia, which appear to be associated with low transmission intensity, early diagnosis and effective treatment, as well as to variable degrees of clinical immunity. Mild-to-moderate clinical and laboratory alterations specific to each parasite species were observed. Interestingly, at this transmission intensity patients infected with either parasite species appear to have a similar risk of developing the levels of severe malaria observed. It is likely that in regions with greater transmission intensity and other socio-economic conditions and more limited health infrastructure, *P. falciparum* patients would progress to severe and lethal cases at a greater rate as compared to *P. vivax* [18,35,56]. Here, no significant differences were observed in clinical and laboratory parameters for patients infected with either *P. vivax* or *P. falciparum*. A prospective study is currently ongoing aimed at a more detailed characterization of malaria complications in hospitalized patients and associated factors, which could contribute to improving MoH policies and strengthening the malaria information system in Colombia.

## Abbreviations

ALT: Alanine aminotransferase
AST: Aspartate aminotransferase
BUN: Blood urea nitrogen
DB: Direct bilirubin
MoH: Ministry of Health
POC: Point-of-care
TB: Total bilirubin
WBC: White blood cells
